# *GRIN2A* Variant in A 3-Year-Old—An Expanding Spectrum?

**DOI:** 10.3390/neurolint13020018

**Published:** 2021-04-29

**Authors:** Ioana Gheța, Raluca Ioana Teleanu, Eugenia Roza, Evelina Carapancea, Oana Vladacenco, Daniel Mihai Teleanu

**Affiliations:** 1 Department of Pediatric Neurology, “Dr Victor Gomoiu” Children’s Hospital, 22102 Bucharest, Romania; ioana.gheta7@gmail.com (I.G.); roza_eugenia@yahoo.com (E.R.); evelina.iachim@gmail.com (E.C.); oanavladacenco@yahoo.com (O.V.); 2 “Carol Davila” University of Medicine and Pharmacy, 020021 Bucharest, Romania; daniel.teleanu@umfcd.ro; 3 “Victor Babes” University of Medicine and Pharmacy, 300041 Timisoara, Romania

**Keywords:** N-methyl-D-aspartate receptor (NMDAR), *GRIN2A* gene, genetic epilepsy, child

## Abstract

Glutamate, the major excitatory neurotransmitter, plays a ubiquitous role in most aspects of normal brain functioning. Its indispensable position is paradoxically doubled by a high excitotoxic potential following disruption of its dynamic equilibrium. Several lines of evidence have suggested the involvement of the glutamatergic N-methyl-D-aspartate receptor (NMDAR) in learning, memory formation, and human cognition. Furthermore, NMDARs play a pivotal role in various neuropsychiatric disorders, recently being identified as an important locus for disease-associated genomic variation. The *GRIN2A* gene encodes the NMDAR’s GluN2A subunit. Genetic alterations of *GRIN2A* result in phenotypic pleiotropy, predisposing to a broad range of epilepsy syndromes, with an elusive and unpredictable evolution and response to treatment. The archetypal *GRIN2A*-related phenotype comprises the idiopathic focal epilepsies (IFEs), with a higher incidence of *GRIN2A* mutants among entities at the more severe end of the spectrum. We report the case of a patient heterozygous for *GRIN2A*, c.1081C>T, presenting with febrile convulsions and later superimposed atonic seizures, expressive language delay, and macrocephaly. As the number of reported *GRIN2A* variants is continuously increasing, the phenotypic boundaries gradually grow faint. Therefore, it is fundamental to maintain an acute critical awareness of the possible genetic etiology of different epilepsy syndromes. So far, therapeutic strategies rely on empirical observations relating genotypes to specific drugs, but the overall success of treatment remains unpredictable. Deciphering the functional consequences of individual *GRIN2A* variants could lead to the development of precision therapeutic approaches for patients carrying NMDAR mutations.

## 1. Introduction

N-methyl-D-aspartate receptors (NMDARs) are ligand-gated cation channels activated by the “Jekyll and Hyde” molecule, glutamate, and co-agonist glycine. They display a heterotetrameric structure, composed of two glycine-binding obligatory GluN1 subunits and two glutamate-binding regulatory subunits of either GluN2 or GluN3 type, expressed in several isoforms (GluN2A–D and GluN3A–B). These different subunits provide the central nervous system with a means of controlling NMDAR properties as a function of the brain region and developmental period. The characteristics of NMDAR-mediated synaptic transmission are dramatically altered during early postnatal development, which is consistent with a developmental change in the composition of the receptors from predominantly GluN1/GluN2B to GluN1/GluN2A. The GluN2B/GluN2A switch is supported by the well-documented postnatal increase in the expression of GluN2A, which confers unique channel properties, mediating the potency of glutamate, the deactivation time course after removal of glutamate, and the high sensitivity to Mg^2+^ block [1,2,3].

Synaptic transmission mediated by NMDARs is critical for the formation and maturation of brain circuits, showing strong involvement in synaptic plasticity, which is the cellular and molecular basis for learning and memory formation. The divergent assembly of the different existing subunits into a diverse array of NMDAR subtypes, each one with unique structural features, allows precise tuning of their physiological roles. As expected from critical structures, NMDA receptors are involved in the pathophysiology of several CNS disorders, such as schizophrenia, Alzheimer’s disease, Parkinson’s disease, Huntington’s disease, epilepsy, and autism. Furthermore, more recently, NMDARs have been identified as being an important locus for disease-associated genomic variation [2,3].

The *GRIN2A* gene maps to chromosome 16p13.2 and encodes the NMDAR GluN2A subunit. *GRIN2A* has been proved to be a gene with a significantly reduced number of missense variants in control subjects compared with the expected number in analogously sized genes, suggesting that *GRIN2A* alterations strongly reduce evolutionary fitness [4]. *GRIN2A* variants have been associated with a broad phenotypic spectrum, the canonical related presentation consisting of speech disorders and epilepsy syndromes. Regarding speech impairment, dysarthria, speech dyspraxia, and both expressive and receptive language delay or regression have been described [5]. As for the epilepsy syndromes, different electroclinical entities have been cited, varying from benign epilepsy with centrotemporal spikes (BECTS) at the mild end of the spectrum to atypical childhood epilepsy with centrotemporal spikes (ACECTS), Landau–Kleffner syndrome (LKS), and epileptic encephalopathy with continuous spike and wave during slow-wave sleep (ECSWS) [6,7,8,9]. In addition, pharmacological manipulations interfering with GluN2A have been associated with short-term memory deficits, defective fear memory formation, and impaired spatial working memory [10,11]. A recent analysis concluded that the *GRIN2A*-related phenotypic spectrum not only comprises well-recognized epilepsy–aphasia disorders but is in fact much broader, ranging from normal or near-normal development to nonspecific epileptic encephalopathy [12].

With an increasing number of identified patients, the *GRIN2A*-associated spectrum might eventually not comply with initially described canonical findings, hindering predictions about the prognosis and therapeutic approach of these cases in the clinical setting. We report one clinical case that we encountered in our practice.

## 2. Case Report

A 32-month-old boy, born to nonsanguineous parents, presented with a history of febrile seizures and macrocephaly. His family history was positive for the presence of febrile convulsions in both his mother and his older sister. The proband was the full-term product of a reportedly pathological pregnancy. His Apgar score was 8/10, but no data could be obtained regarding his birth head circumference. He displayed normal psychomotor development except for a delayed acquisition of language, being able to produce around 8 to 10 words at the time of presentation and lacking the ability to produce phrases.

The onset of seizures was at 25 months of age, with a sole episode depicted as unresponsiveness followed by tonic generalized muscle contraction, with eyelid retraction and upward eye deviation with a total duration of around 2 min, in association with a fever of 38 °C. Three months later, a second seizure occurred, also at the onset of an acute febrile illness, no recurrence being noted within one fever episode. Neurological evaluation and EEG recording at that time revealed no pathological findings. Regardless, another two similar episodes followed, and the child was started on valproic acid.

Following the recurrence of febrile seizures in spite of the antiseizure treatment, at 32 months of age, he presented to our clinic. The neurological examination noted the presence of macrocephaly and expressive language delay, and the EEG recording showed bilateral frontocentral spike-and-wave discharges. Valproic acid was titrated to a therapeutic dose, with good clinical response.

Neuroimaging revealed discrete asymmetry of the lateral ventricles and the presence of an inflammatory process of the left mastoid air cells. A potentially genetic etiology was considered, given the positive family history. Sequence analysis using an epilepsy gene panel identified a heterozygous variant of *GRIN2A*, c.1081C>T, p.(Leu361=), classified as a variant of uncertain significance (VUS).

A follow-up sleep EEG at 33 months of age revealed the presence of random multifocal myoclonic movements of the extremities with corresponding generalized discharges of sharp waves. Nitrazepam was added to his antiepileptic drug (AED) regimen with improvement in frequency and amplitude of sleep myoclonus. Over time, EEG showed normal background, with rare, irregular runs of frontal sharp wave discharges during sleep, slightly more prominent on the left.

After 5 months without any documented seizures, at 3 years of age, the child presented three afebrile seizures with loss of muscle tone resulting in abrupt falling, with spontaneous recovery in 10–12 s. Postictal state was characterized by unconsolable crying and abdominal pain. Furthermore, iteration of the initial type of seizures was also noted. Serum valproate levels were found to be low, so the dose was adjusted accordingly. Nevertheless, seizures failed to cease, and a second determination of serum valproate concentration still revealed inadequate levels. EEG showed sleep-activated left frontocentral epileptiform discharges. Thereafter, nitrazepam dose was increased, which led to seizure remission at 3 years and 2 months of age.

Subsequent EEG recordings demonstrated a relatively stable morphology over time, with bilateral centrotemporal spike-and-wave discharges, more prominent on the left side, without evidence of continuous spike and wave during sleep. The proband remained seizure-free, but he continued to display expressive language impairment and severe dyslalia at 4.5 years of age despite appropriate speech and language therapy.

## 3. Discussion

The notorious association between *GRIN2A* mutations and epilepsy–aphasia syndromes (EAS) has been widely acknowledged, providing the clinician with an acute clinical awareness of the possible etiology of an easily recognizable phenotypic spectrum [5]. Nevertheless, an increasing number of authors have reported heterogeneous epileptic phenotypes in patients with *GRIN2A* variants, stressing the importance of maintaining a high level of suspicion regarding the complex interaction of various genetic, environmental, and epigenetic factors determining seizure presentation. Hasaerts et al. described an extensive range of different clinical and electrical seizures, including focal clonic seizures, unilateral and generalized spasms, and tonic seizures in an infant with a *GRIN2A* mutation who displayed severe developmental delay and stereotyped behavior [13]. One report mentions a pseudo-Dravet phenotype in a patient with a missense mutation in the *GRIN2A* gene, mentioning the occurrence of focal clonic, atonic, myoclonic, and tonic–clonic seizures in association with interictal left temporal spike-and-wave paroxysms, not modified by sleep [14]. Furthermore, Fernández-Marmiesse et al. described two siblings carrying a missense mutation in *GRIN2A,* presenting with a neurodevelopmental and movement disorder, without any epileptiform abnormalities [15].

Our patient presented with febrile seizures, and later afebrile superimposed atonic seizures and sleep myoclonus. He also displayed expressive language delay with severe dyslalia and macrocephaly. His EEG recordings stabilized to a pattern of interictal bilateral, left-side predominant, centrotemporal spike-and-wave discharges, suggestive of rolandic epilepsy. Genetic analysis was performed in this particular case prior to the evolution of the patient’s phenotype, when he solely manifested febrile convulsions, dictated by the presence of a positive family history of febrile seizures. DNA sequence analysis revealed a heterozygous synonymous variant of *GRIN2A*, c.1081C>T, classified as a variant of uncertain significance (VUS).

It has been theorized that *GRIN2A* mutations can lead to different NMDAR properties being altered, including glutamate and glycine potency, sensitivity to Mg^2+^, extracellular protons and Zn^2+^, agonist-evoked current amplitude, single-channel opening time and open probability, and total and surface receptor expression levels. Furthermore, changes in the receptor properties might sometimes be contradictory, leading, for example, to inconsistencies in agonist sensitivity or response to allosteric modulators [15,16,17,18,19,20].

In our patient, febrile seizures were the harbinger of the consequent epileptic syndrome. Recent work provides genetic evidence that altered homeostasis of brain Zn^2+^ (i.e., low synaptic Zn^2+^) is associated with increased cellular excitability and febrile seizure susceptibility [20]. Extracellular Zn^2+^ ions are able to specifically inhibit GluN2A-containing NMDARs due to an interaction site on the GluN2A N-terminal domain. Two *GRIN2A* mutations, P79R and R370W, were found to alter GluN2A-mediated high-affinity zinc inhibition. Both variants resulted in overlapping clinical symptoms within the spectrum of rolandic epilepsies, although in vitro analysis demonstrated puzzling opposite functional consequences (decreased and increased zinc inhibition, respectively) [2]. In silico analysis of our patient’s *GRIN2A* c.1081C>T variant could not provide enough data to support or rule out pathogenicity. In vitro approaches represent an important tool for investigating the functional relevance of a gene mutation. They allow testing of functional properties regarding ligand affinity, downstream signaling, and ion current changes. In vitro analysis was not addressed in our proband; however, given the distinct seizure semiology, one might speculate that the zinc–NMDAR interaction assessment might prove worthwhile.

Our patient’s electroencephalography findings were typical for the rolandic epilepsy (RE) spectrum. However, he did not exhibit focal sensorimotor seizures as one could have expected judging from his EEG abnormalities. Instead, he developed atonic seizures following the initial febrile convulsions. Albeit uncommon, brief atonic seizures leading to frequent falls, known as epileptic negative myoclonus (ENM), represent one of the most notorious features of atypical childhood epilepsy with centrotemporal spikes (ACECTS) [21]. Polygraphic monitoring allowing the demonstration of transient interruptions of a tonic EMG activity and simultaneous EEG epileptic potential would have been needed for the definite interpretation of the patient’s atonic seizures as ENM. Furthermore, negative myoclonus usually occurs after a latency period of months to years marked by typical rolandic events, often as a complication of AED treatment, particularly carbamazepine, which was not consistent with our patient’s history. However, Watemberg et al. reported on epileptic negative myoclonus as the initial manifestation of childhood epilepsy with centrotemporal spikes in the absence of typical rolandic seizures [22].

Treatment with sodium valproate and nitrazepam eventually led to seizure freedom, but the speech impairment persisted despite appropriate speech and language therapy. Nonetheless, whether the speech phenotype was solely derived from the genetic *GRIN2A* alteration or was prejudiced by the long-term treatment with nitrazepam remains a valid question.

Judging from the dramatically increasing number of known NMDAR subunit mutations in epilepsy, NMDARs can be regarded as important molecular targets for epilepsy therapy [23,24]. Seizures in mild epileptic types, such as BECTS, are usually easily controlled with standard antiseizure medication. Von Stülpnagel and colleagues reported that *GRIN2A* patients demonstrated a favorable response to commonly used drugs in idiopathic focal epilepsy, especially valproic acid, sulthiame, clobazam, and steroids [20]. However, more severe epilepsy syndromes present with intractable seizures, representing promising candidates for treatment with allosteric modulators of the NMDAR. In patients with refractory epileptic encephalopathy and *GRIN2A* mutations, memantine, a noncompetitive antagonist of the NMDAR, has been successfully used to control seizures, either alone or in combination with standard antiseizure medication [6,12,15,25,26,27]. Furthermore, it has recently been suggested that immunotherapy may lead to clinical and electroencephalographic improvement in patients with *GRIN*-related epileptic encephalopathies, addressing the potential role of autoimmunity in *GRIN*-related disorders [28]. These recent findings might strengthen the previously proposed association between anti-NMDAR encephalitis and *GRIN* mutations. The reversible depletion of receptors in anti-NMDAR encephalitis has been shown to substantially enhance the excitability of the motor cortex, supposedly pointing to overactivity of glutamatergic pathways, being compatible with models of genetic reduction of NMDAR. Similarities in terms of seizure semiology and origination, as well as language impairment, have previously been emphasized, and with novel *GRIN2A* phenotypes comprising movement disorders and sleep disturbances being described, the gap between the two clinical entities narrows [15,29,30]. Further work is required to elucidate the molecular mechanisms underlying distinct phenotypes and to formulate the prerequisites for the development of precision medicine approaches for patients carrying NMDAR mutations.

## 4. Conclusions

Correlating epileptic phenotype to genotype proves to be difficult in the context of polygenic interactions and multiple environmental and epigenetic factors involved in the expression of seizures. At present, existing data on *GRIN2A* mutations offer a rather confusing description of the phenotypic spectrum, as the number of reported variants is continuously increasing. The notoriously quoted *GRIN2A*-related epilepsy–aphasia spectrum, although still at the center of the clinical picture, only accounts for a fragment of the presentations. Therefore, a high level of suspicion should be maintained, extending the spectrum of genes to be studied in epilepsy.

A therapeutic approach to genetic epilepsies primarily relies on empirical observations relating genotypes to response to specific drugs. Following the impressive advances in unraveling the molecular mechanisms underlying the clinical manifestations of the disease in the individual patient, future prevailing paradigms will involve reverse sequencing, starting with the minute characterization of the functional consequences of the pathogenic variants and identifying precision therapeutic approaches.

## References

[1] Feldman D.E., Knudsen E.I. (1998). Experience-dependent plasticity and the maturation of glutamatergic synapses. Neuron.

[2] Franchini L., Carrano N., Di Luca M., Gardoni F. (2020). Synaptic GluN2A-Containing NMDA Receptors: From Physiology to Pathological Synaptic Plasticity. Int. J. Mol. Sci..

[3] Hansen K.B., Yi F., Perszyk R.E., Furukawa H., Wollmuth L.P., Gibb A.J., Traynelis S.F. (2018). Structure, function, and allosteric modulation of NMDA receptors. J. Gen. Physiol..

[4] Lek M., Karczewski K.J., Minikel E.V., Samocha K.E., Banks E., Fennell T., O’Donnell-Luria A.H., Ware J.S., Hill A.J., Cummings B.B. (2016). Analysis of protein-coding genetic variation in 60,706 humans. Nature.

[5] Yang X., Qian P., Xu X., Liu X., Wu X., Zhang Y., Yang Z. (2018). GRIN2A mutations in epilepsy-aphasia spectrum disorders. Brain Dev..

[6] Xu X.-X., Luo J. (2017). Mutations of N-Methyl-D-Aspartate Receptor Subunits in Epilepsy. Neurosci. Bull..

[7] Myers K.A., Scheffer I.E., Adam M.P., Ardinger H.H., Pagon R.A., Wallace S.E., Bean L.J.H., Mirzaa G., Amemiya A. (2016). GRIN2A-Related Speech Disorders and Epilepsy. GeneReviews^®^.

[8] Lesca G., Møller R.S., Rudolf G., Hirsch E., Hjalgrim H., Szepetowski P. (2019). Update on the genetics of the epilepsy-aphasia spectrum and role of GRIN2A mutations. Epileptic Disord..

[9] Lal D., Steinbrücker S., Schubert J., Sander T., Becker F., Weber Y.G., Lerche H., Thiele H., Krause R., Lehesjoki A.-E. (2015). Investigation of GRIN2A in common epilepsy phenotypes. Epilepsy Res..

[10] Bannerman D.M., Niewoehner B., Lyon L., Romberg C., Schmitt W.B., Taylor A., Sanderson D.J., Cottam J., Sprengel R., Seeburg P.H. (2008). NMDA Receptor Subunit NR2A Is Required for Rapidly Acquired Spatial Working Memory But Not Incremental Spatial Reference Memory. J. Neurosci..

[11] Zhang X.-M., Yan X.-Y., Zhang B., Yang Q., Ye M., Cao W., Qiang W.-B., Zhu L.-J., Du Y.-L., Xu X.-X. (2015). Activity-induced synaptic delivery of the GluN2A-containing NMDA receptor is dependent on endoplasmic reticulum chaperone Bip and involved in fear memory. Cell Res..

[12] Strehlow V., Heyne H.O., Vlaskamp D.R.M., Marwick K.F.M., Rudolf G., Bellescize J., Biskup S., Brilstra E.H., Brouwer O.F., Callenbach P.M.C. (2019). GRIN2A-related disorders: Genotype and functional consequence predict phenotype. Brain.

[13] Hasaerts D., Jonckheere A., Jansen A., De Meirleir L., Lederer D. (2015). P40–2775: Unusual presentation in a child with a mutation in GRIN2A. Eur. J. Paediatr. Neurol..

[14] Aguilar-Castillo M., Petersen N.C., Romero T.D.H., Spuch A., De Vicente G., Torres-Puchol F.G., Serrano-Castro P. (2019). Epileptic encephalopathy with phenotype pseudo-Dravet and missense mutation in the GRIN2A gene. Clin. Chim. Acta.

[15] Fernández-Marmiesse A., Kusumoto H., Rekarte S., Roca I., Zhang J., Myers S.J., Traynelis S.F., Couce M.L., Gutiérrez-Solana L., Yuan H. (2018). A novel missense mutation in GRIN2A causes a nonepileptic neurodevelopmental disorder. Mov. Disord..

[16] Pierson T.M., Yuan H., Marsh E.D., Fuentes-Fajardo K., Adams D.R., Markello T., Golas G., Simeonov D.R., Holloman C., Tankovic A. (2014). GRIN2Amutation and early-onset epileptic encephalopathy: Personalized therapy with memantine. Ann. Clin. Transl. Neurol..

[17] Swanger S.A., Chen W., Wells G., Burger P.B., Tankovic A., Bhattacharya S., Strong K.L., Hu C., Kusumoto H., Zhang J. (2016). Mechanistic Insight into NMDA Receptor Dysregulation by Rare Variants in the GluN2A and GluN2B Agonist Binding Domains. Am. J. Hum. Genet..

[18] Marwick K.F., Skehel P.A., Hardingham G.E., Wyllie D.J.A. (2019). The human NMDA receptor GluN2AN615K variant influences channel blocker potency. Pharmacol. Res. Perspect..

[19] Carvill G.L., Regan B.M., Yendle S.C., O’Roak B.J., Lozovaya N., Bruneau N., Burnashev N., Khan A., Cook J., Geraghty E. (2013). GRIN2A mutations cause epilepsy-aphasia spectrum disorders. Nat. Genet..

[20] Gao K., Tankovic A., Zhang Y., Kusumoto H., Zhang J., Chen W., XiangWei W., Shaulsky G.H., Hu C., Traynelis S.F. (2017). A de novo loss-of-function GRIN2A mutation associated with childhood focal epilepsy and acquired epileptic aphasia. PLoS ONE.

[21] Reid C.A., Hildebrand M.S., Mullen S.A., Hildebrand J.M., Berkovic S.F., Petrou S. (2016). Synaptic Zn^2+^ and febrile seizure susceptibility. Br. J. Pharmacol..

[22] Watemberg N., Leitner Y., Fattal-Valevski A., Kramer U. (2009). Epileptic Negative Myoclonus as the Presenting Seizure Type in Rolandic Epilepsy. Pediatr. Neurol..

[23] Vieira M., Nguyen T.A., Wu K., Badger J.D., Collins B.M., Anggono V., Lu W., Roche K.W. (2020). An Epilepsy-Associated GRIN2A Rare Variant Disrupts CaMKIIα Phosphorylation of GluN2A and NMDA Receptor Trafficking. Cell Rep..

[24] Perucca P., Perucca E. (2019). Identifying mutations in epilepsy genes: Impact on treatment selection. Epilepsy Res..

[25] Von Stülpnagel C., Ensslen M., Møller R.S., Pal D., Masnada S., Veggiotti P., Piazza E., Dreesmann M., Hartlieb T., Herberhold T. (2017). Epilepsy in patients with GRIN2A alterations: Genetics, neurodevelopment, epileptic phenotype and response to anticonvulsive drugs. Eur. J. Paediatr. Neurol..

[26] Amador A., Bostick C.D., Olson H.E., Peters J., Camp C.R., Krizay D., Chen W., Han W., Tang W., Kanber A. (2020). Modelling and treating GRIN2A developmental and epileptic encephalopathy in mice. Brain.

[27] Mir A., Qahtani M., Bashir S. (2020). GRIN2A-Related Severe Epileptic Encephalopathy Treated with Memantine: An Example of Precision Medicine. J. Pediatr. Genet..

[28] Hausman-Kedem M., Menascu S., Greenstein Y., Fattal-Valevski A. (2020). Immunotherapy for GRIN2A and GRIN2D-related epileptic encephalopathy. Epilepsy Res..

[29] Manto M., Dalmau J., Didelot A., Rogemond V., Honnorat J. (2010). Afferent facilitation of corticomotor responses is increased by IgGs of patients with NMDA-receptor antibodies. J. Neurol..

[30] Dalmau J., Lancaster E., Martinez-Hernandez E., Rosenfeld M.R., Balicegordon R.J. (2011). Clinical experience and laboratory investigations in patients with anti-NMDAR encephalitis. Lancet Neurol..

